# Some Observations on the Use of Acetozone

**Published:** 1906-12

**Authors:** B. P. Wilson

**Affiliations:** Reddick, Fla.


### SOME OBSERVATIONS ON THE USE OF ACETOZONE.

BY B. P. WILSON, M.D.,

REDDICK, FLA.

While my opportunities for testing the virtues of acetozone have been limited, owing to the fact that we see but little of the diseases in this section for which it is recommended, I feel safe in saying that the practitioner who treats typhoid fever or pneumonia without giving his patient the benefit of a thorough course of acetozone has failed to do his whole duty and has lessened the chances of one human being's recovery. In one case of typhoid fever that I saw for the first time at the end of the second week, when the temperature ranged from 103½° to 105½°, acetozone so altered the condition of things that it thereafter ranged from 99½° to 101° for three weeks, when convalescence was established.

I administered acetozone to an old lady seventy years of age, who had a well-marked case of pneumonia involving the upper lobe of the right lung, and in which the prognosis was grave. Thereafter her temperature ranged from 101½° to 102½° while previously it had ranged two degrees higher.

The physician who secures and maintains a thoroughly aseptic condition of the primæ viæ in his cases of pneumonia will find that it adds to the chances of recovery as much or more than any one other thing in the way of treatment.

---

IN children, in cases of peritonitis of unknown origin, examine for gonorrheal vulvo-vaginitis.—*American Journal of Surgery.*

